# Comparison of ARIMA and GM(1,1) models for prediction of hepatitis B in China

**DOI:** 10.1371/journal.pone.0201987

**Published:** 2018-09-04

**Authors:** Ya-wen Wang, Zhong-zhou Shen, Yu Jiang

**Affiliations:** School of Public Health, Chinese Academy of Medical Sciences & Peking Union Medical College, Beijing, China; Columbia University, UNITED STATES

## Abstract

**Background:**

Hepatitis B virus (HBV) infection is a major public health threat in China for China has a hepatitis B prevalence of more than one million people in 2017 year. Disease incidence prediction may help hepatitis B prevention and control. This study intends to build and compare 2 forecasting models for hepatitis B incidence in China.

**Methods:**

Autoregressive integrated moving average (ARIMA) model and grey model GM(1,1) were adopted to fit the monthly incidence of hepatitis B in China from March 2010 to October 2017. The fitting and forecasting performances of the 2 models were evaluated. The better one was adopted to predict the incidence from November 2017 to March 2018. Database was built by Excel 2016 and statistical analysis was completed using R 3.4.3 software.

**Results:**

Descriptive analysis showed that the incidence of hepatitis B in China has seasonal variation and has shown a downward trend from 2010 to 2017. We selected the ARIMA (3,1,1) (0,1,2)_12_ model among all the ARIMA models for it has the lowest AIC value. Model expression of GM (1,1) was *X*^(1)^ (*k* + 1) = 3386876.7478*e*^0.0249*k*^ − 3289206.7428. The root mean square error (RMSE), mean absolute error (MAE) and mean absolute percentage error (MAPE) of ARIMA(3,1,1)(0,1,2)_12_ model were lower than GM(1,1) model on fitting part and forecasting part. According to the forecast results, the incidence may have a slight fluctuation during the following months.

**Conclusions:**

The ARIMA model showed better hepatitis B fitting and forecasting performance than GM(1,1) model. It is a potential decision supportive tool for controlling hepatitis B in China before a predictive hepatitis B outbreak.

## Introduction

Hepatitis B, an infectious disease caused by Hepatitis B virus (HBV) infection, is still a serious public health issue despite having available effective vaccines [1]. As many as 2 billion people have been infected around the world and more than 240 million people are chronic carriers [2]. Progressive liver diseases will develop in most chronic HBV infected people, such as cirrhosis, liver failure, and hepatocellular carcinoma (HCC), all of which has high mortality rate [3]. According to a survey of 50 countries, the prevalence of hepatitis B in China was higher than most of European and American countries [4] although a decrease trend has been seen in the past decade. Due to the large population, even a low incidence rate means a huge crowd of hepatitis B infected people, which reduces life quality and aggravates the social burden. Thus, an appropriate prediction may offer some suggestions and provide references in hepatitis B prevention and control.

The sense of diseases prediction varies from different usage. Generally, historical data is adopted to create model and predict the current development trend. The predicted value is the compared with the actual value to judge whether the disease managements taken in the past, such as vaccine, are effective. Besides, develop a model with current data and predict the future trend of disease. If the real value exceeds the upper limit of the predicted value, an outbreak should be prevented.

Currently, several mathematical methods are applied in disease incidence prediction such as linear regression, artificial neural network and grey model. The ARIMA model is commonly used in infectious disease time series prediction, especially for series that has a cyclic or repeating pattern. The model was conceived for economics applications but well applied in medical field nowadays. The principle of the model contains filtering out the high-frequency noise in the data, detecting local trends based on liner dependence and forecasting the develop trends [5]. Despite its high predictive performance, the model has some limitations which decrease its scope of application. The model assumes a linear relationship between the dependent and independent variables while the actual data often present non-linear relationships. Besides, the model assumes that the mean and variance of response series are independent of time, which means stationary [6]. Thus, more than one model should be tested to choose a better one.

Grey prediction is another method to predict time series which has different set of principles than ARIMA model. It focuses on grey system and was established by Prof. Deng in the 1980s [7]. Grey system is different from white system and black system. White system means certain problems and all information is known and black system means that nothing is known about the data [8]. Grey system means uncertain problems, incomplete information, often with small sample size and fuzzy mathematics to handle. For incidence of infectious disease, all the information we know is the incidence and time. Since there are other unknown influencing factors, the grey model (GM) might be appropriate [9]. GM (1,1) is one of the basic model of grey prediction, and the model expression means first order equation and single variable[10]. A wide range of real-world problems have been tested with GM model such as engineering problems, energy consumption, environmental problem, disease forecasting and so on [11–15].

In this study, ARIMA model and GM (1,1) model based on the monthly incidence of hepatitis B in China were built and compared. The model building and comparison intends to give some suggestions on the model chosen and the predicted values may offer references for hepatitis B prevention.

## Materials and method

### Materials source

The monthly incidence data of hepatitis B in China from March 2010 to October 2017 were collected from the official website of National Health Commission of the People’s Republic of China (Ministry of Health). Since GM (1,1) model has less requirement of data and according to some existing studies, five to ten samples are enough to build grey model, we use different sample size to build these two models. Data from March 2010 to May 2017 were used to build the ARIMA model and data from August 2016 to May 2017 were used to develop the GM (1,1) model. Data from June to October 2017 were used to evaluate these models’ forecasting performance.

### ARIMA model

ARIMA model contains auto regressive (AR) model, moving average (MA) model, seasonal autoregressive integrated moving average (SARIMA) model and etc. The model is expressed as ARIMA (p, d, q)(P,D,Q)_S_ generally, *p* means the order of auto-regression, *d* means the degree of trend difference, *q* means the order of moving average, *P* means the seasonal auto-regression lag, *D* means the degree of seasonal difference, *Q* means the seasonal moving average, *s* means the length of the cyclical pattern [16]. Time series stationary, parameter estimation, model check and prediction were done to establish the ARIMA model [17, 18].

#### Time series stationary

Since ARIMA model requires stationary time series, which means the time series shows no fluctuation or periodicity with time. The Augmented Dickey-Fuller (ADF) unit-root test could help estimating whether the time series is stationary or not. Log transformation and differences are preferred ways to stabilize the time series [19], seasonal and non-seasonal differences were adopted to stabilize the term trend and periodicity in this study.

#### Parameter estimation

Parameters of ARIMA model were estimated by autocorrelation function (ACF) graph and partial autocorrelation (PACF) graph. Automatic identification and artificial estimation were adopted in this study. “auto.arima()” command in R software was adopted first to automatically identify the model parameters. Then ACF and PACF were employed to identify p, q and P, Q.

#### Model evaluation

Models of varying orders of p, q and P, Q were tested through Box-Jenkibs Q test [20]. All the models that passed the residual test (show a white noise sequence) were compared using Akaike information criterion (AIC) so that a best model can be found. In this study, we used the incidence of Hepatitis B from March 2010 to October 2017 to build and test the ARIMA model. The model’s fitting and prediction power were evaluated by comparing the theoretical values with real values.

### GM (1,1) model

Incidence data from August 2016 to May 2017 were used to build the GM (1,1) model and data from September 2016 to May 2017 were used as back substitution to test the fitting power. Forecasting performance was test by predictive values and actual values form June to October 2017. The steps of building a GM (1,1) model include original time sequence, accumulated generating operation (AGO), adjacent neighbor means, whitenization equation and inverse AGO [21, 22].

The nonnegative original time sequence x^(0)^ and AGO time series x^(1)^ showed as:

$$x^{\left(0\right)}=(x^{\left(0\right)}(1),x^{\left(0\right)}(2),\ldots x^{\left(0\right)}(n))$$ (1)

$$x^{\left(1\right)}=(x^{\left(1\right)}(1),x^{\left(1\right)}(2),\ldots x^{\left(1\right)}(n))$$ (2)

n is the sample size of the data.

Adjacent neighbor means. Calculating the mean of AGO time series and showed as:

$$y^{\left(1\right)}=\frac{1}{2}[x^{\left(1\right)}(k)+x^{\left(1\right)}(k-1)]$$ (3)

k = 2,3…,n.

The whitenization equation was showed as:

$$\frac{dx^{\left(1\right)}}{dt}+ax^{\left(1\right)}=u$$ (4)

In this equation, *a* is developing coefficient and *u* is control variable. These are two parameters of GM(1,1) model. In addition, a is an assistant to estimate the GM(1,1) model’s prediction length (Table 1).

**Table 1:** Developing coefficient and prediction length.

Developing Coefficient *a*	Prediction Length
-a≤0.3	Medium- and long-term prediction
0.3<-a≤0.5	Short-term prediction
0.5<-a≤1.0	Modified model to predict
1.0≤-a	Not suitable for grey prediction model

Inverse AGO was done to develop GM(1,1) model and showed as:

$$x^{\left(1\right)}(k+1)=[x^{\left(1\right)}(0)-\frac{u}{a}]e^{\left(-ak\right)}+\frac{u}{a}$$ (5)

Test of GM(1,1) model:

1. Coincidence rate: The ratio of the predicted value and actual value, expressed as percentage.
2. The post-test ratio (C): C = Se/Sx. *Se* means the standard deviation of residual series and *Sx* means the standard deviation of original time series. The value reflects the concentration degree of the difference between predicted value and actual value. The smaller the C is, the more concentrated the difference is.
3. Small error probability (P): Calculating the difference between residual and it’s mean and P is the ratio of the difference to 0.6475Sx. The greater the P is, the closer the difference to 0.6475Sx. P and C are combined to evaluate the fitting effect of GM(1,1) model (Table 2).
4. Relative error: The relative error of an optimal model should less than 5% generally, but it is still acceptable if the relative error is higher than 5% but less than 20%.

**Table 2:** Accuracy evaluation criteria of GM(1,1) model.

Accuracy Criteria	P	C
High	0.95≤P	C≤0.35
Good	0.80≤P<0.95	0.35<C≤0.50
Qualified	0.70≤P<0.80	0.50<C≤0.65
Disqualified	P<0.70	0.65<C

### Forecast accuracy access

Three indexes were employed in accessing model fitting and forecasting efficiency: RMSE, MAE and MAPE [23]. These three indexes are defined as:

$$RMSE=\sqrt{\frac{\sum_{i=1}^{n}(X_{i}-{\hat{X}}_{i}{)}^{2}}{n}}$$ (6)

$$MAE=\frac{\sum_{i=1}^{n}\left|X_{i}-{\hat{X}}_{i}\right|}{n}$$ (7)

$$MAPE=\frac{\sum_{i=1}^{n}\frac{\left|X_{i}-{\hat{X}}_{i}\right|}{X_{i}}\times 100}{n}$$ (8)

*X*_*i*_ is the actual value, ${\hat{X}}_{i}$ is the predict value and n is the number of observation.

### Data processing and analysis

Excel 2016 was used to build the database of monthly incidence of Hepatitis B in China and R 3.4.3 software was adopted to develop the ARIMA model and GM(1,1) model. Significant level is 0.05.

### Ethics

Since no primary data collection was undertaken, no patient or public was involved, no formal ethical assessment or informed consent was required. All data were collected from the official website and all data were fully anonymized.

## Results

### Trends in hepatitis B in China

**Fig 1:**
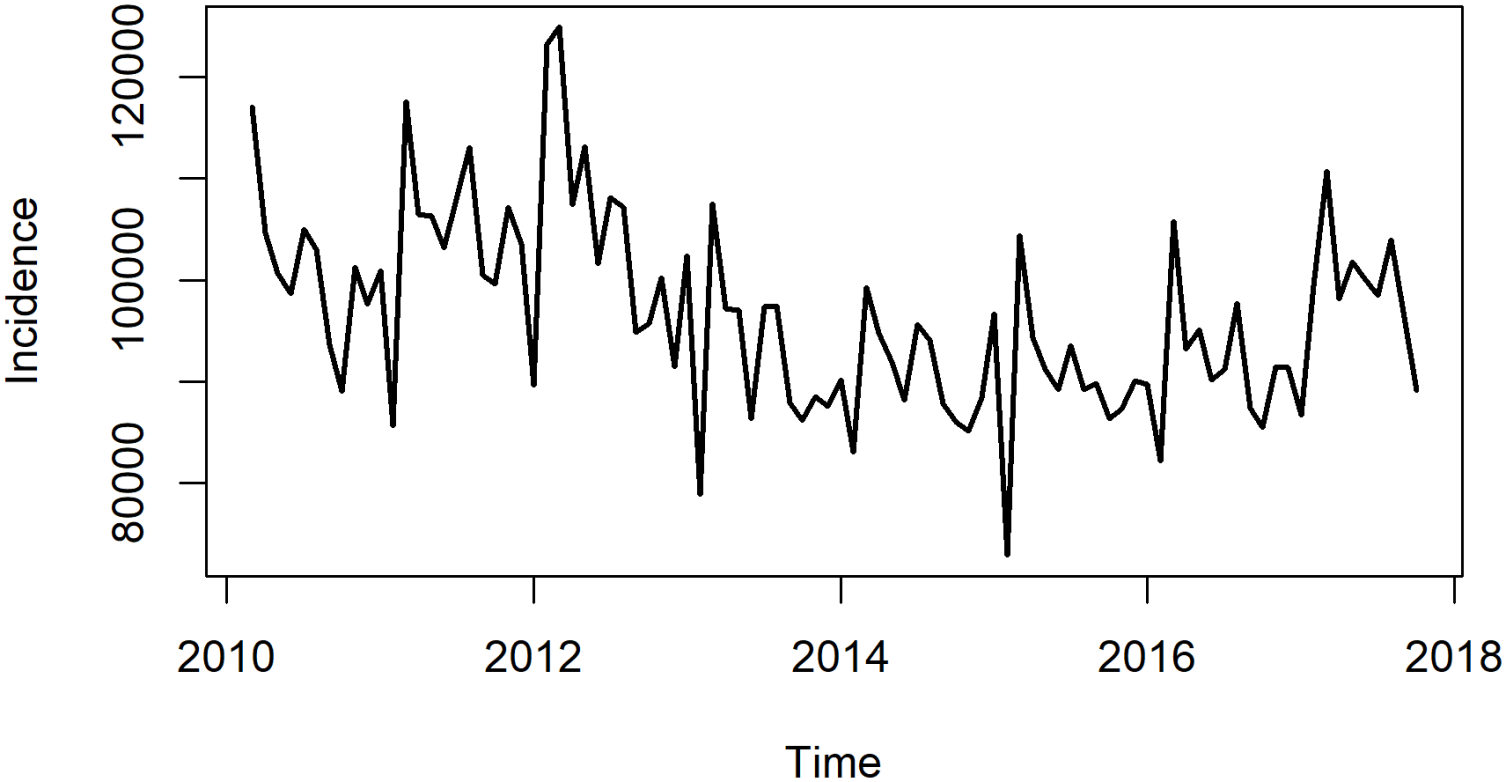
Monthly incidence of hepatitis B in China from March 2010 to October 2017.

A total of 87 numbers were collected to develop ARIMA model and 10 were used to develop GM(1,1) model. Fig 1 showed that the overall incidence of Hepatitis B in China presented a downward trend from 2010 to 2017. The incidence went down from 2010 to 2014 and had a slight rising trend from then on. In a year, January and February showed lowest value and followed by a rapid rise. A strong periodicity can be seen.

### ARIMA model

**Fig 2:**
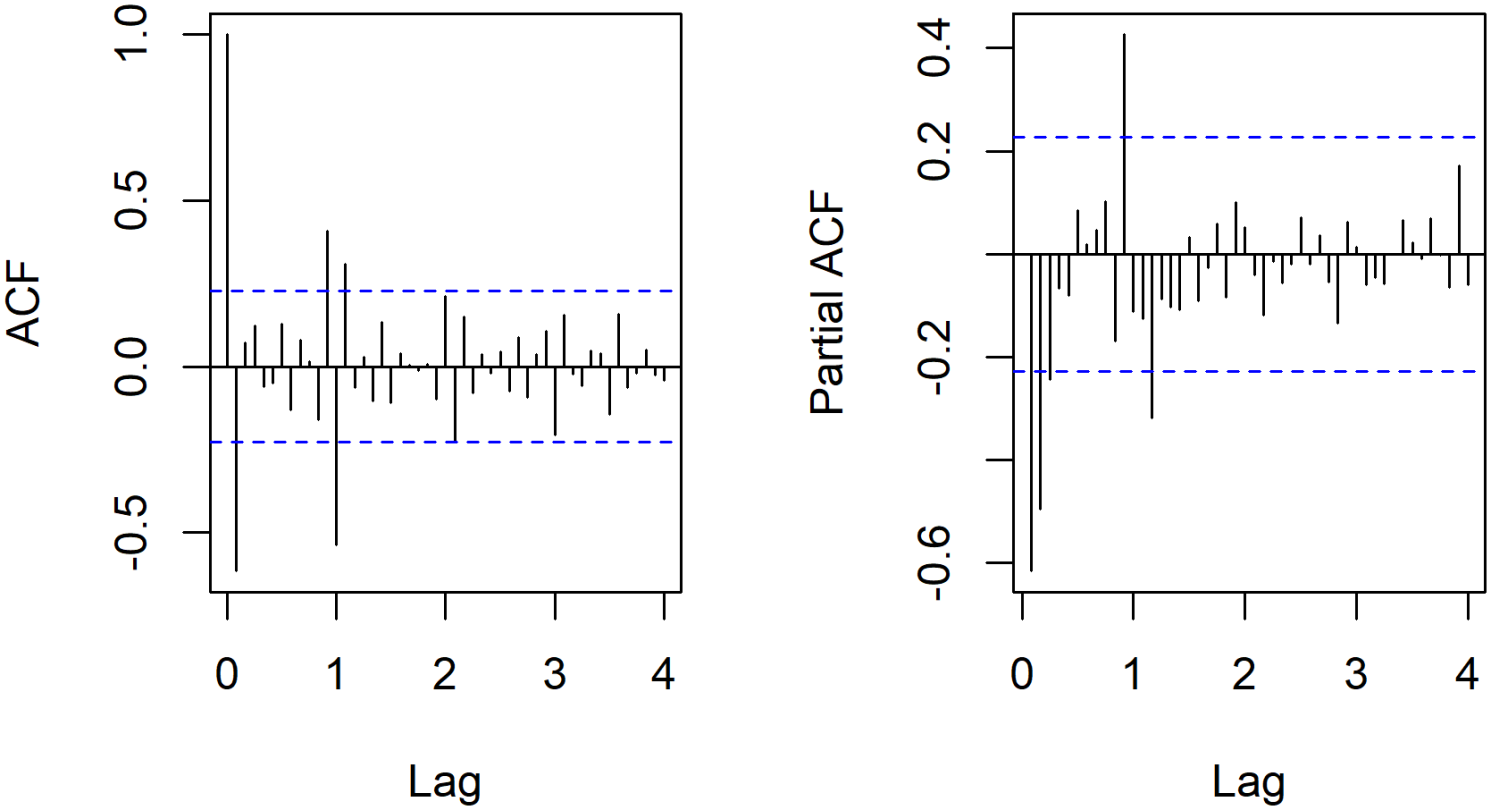
The ACF graph and PACF graph of differenced hepatitis B incidence series.

The incidence data of Hepatitis B in China from March 2010 to May 2017 showed a non-stationary trend with time. First trend difference (d = 1) and seasonal difference (D = 1) were done to eliminate numerical instabilities. ADF test (Table 3) showed statistically significant (p = 0.01). Then the ACF graph and PACF graph (Fig 2) were done to help estimate the parameters.

**Table 3:** The ADF test of the differenced time series.

Covariate	t-Statistic	p-value
ADF test statistic	-5.6842	0.01
1% level statistic	-2.6	—
5% level statistic	-1.95	—
10% level statistic	-1.61	—

During 1 circle, ACF declined to 0 after lag 1 and PACF was at lag3, thus p = 3, q = 1. During 4 circles, ACF declined at the end of the first circle (lag 12) but close to 0, thus Q = 1 or 2. PACF was 0 at lag 12, thus P = 0. P = 1 was tested to make the results more dependable. D = d = 1. In addition, “auto.arima()” of R 3.4.3 software was used to recognize parameters automatically. So, 5 models were combined. The results of residual test and AIC values are shown in Table 4.

**Table 4:** Residual test and AIC.

Combined model	Lag				AIC
	Lag 6	Lag 12	Lag 18	Lag 24	
ARIMA(2,1,0)(1,1,0)_12_	0.6901	0.7277	0.8461	0.6672	1520.05
ARIMA(3,1,1)(0,1,1)_12_	0.9198	0.6645	0.8357	0.9601	1516.21
ARIMA(3,1,1)(1,1,1)_12_	0.3835	0.3325	0.4517	0.6976	1517.22
ARIMA(3,1,1)(0,1,2)_12_	0.8507	0.6167	0.7675	0.9029	1515.24
ARIMA(3,1,1)(1,1,2)_12_	0.8267	0.6285	0.7784	0.8711	1516.55

According to Table 4, all models meet the requirement of white noise of residual time series, so the AIC values were compared. Automatically recognized model ARIMA(2,1,0)(1,1,0)_12_ did not meet the criterion because of the highest AIC value. ARIMA(3,1,1)(0,1,2)_12_ had the lowest AIC and was selected as the best ARIMA model of this study.

### GM(1,1) model

Data from August 2017 to May 2017 was employed to create the GM(1,1) model and data from June to October 2017 was used to test the model’s forecasting performance. The evolution parameter *a* was -0.0249 and gray variable *u* was 82039.98. The equation was *X*^(1)^ (*k* + 1) = 3386876.7478*e*^0.0249*k*^ − 3289206.7428, k is the number of time series. The post-test ratio C was 0.4622 and small error probability P was 0.9000, which means good prediction accuracy.

### Model comparison

**Fig 3:**
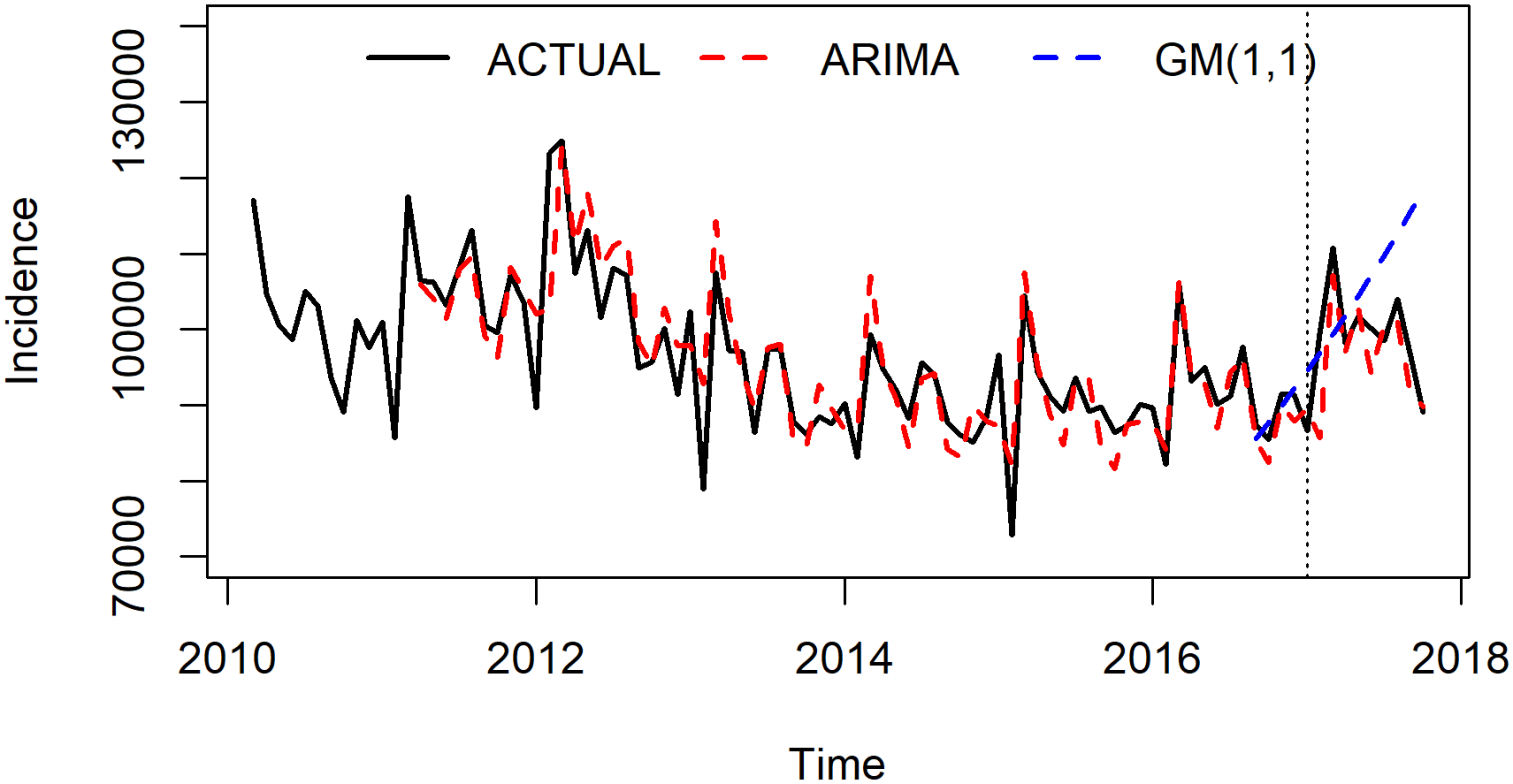
The observed hepatitis B incidence and fitting and forecasting values simulated by ARIMA and GM(1,1) models.

ARIMA(3,1,1)(0,1,2)_12_ and GM(1,1) model were adopted to forecast the number of Hepatitis B from June to October 2017. Predictions were compared with the actual values to test the model’s forecasting effect. Three indicators were applied to evaluate the models’ performance and the results showed that ARIMA model was better than GM(1,1) model in fitting and forecasting part (Table 5). Fig 3 shows the fitting and forecasting curves of these two models. The observed Hepatitis B incidence and fitting and forecasting values of ARIMA model and GM (1,1) model were divided into fitting part and forecasting part by a vertical dashed line, the left was the fitting stage, and the right was the forecasting stage. ARIMA model fitted and predicted the seasonal fluctuation well while GM (1,1) model could not suitably recognize it.

**Table 5:** The fitting and forecasting performance of the two models.

Model	Fitting part			Forecasting part		
	MAPE	MAE	RMSE	MAPE	MAE	RMSE
ARIMA	3.7224	3522.8090	4957.9215	3.3896	3358.3000	3849.7170
GM (1,1)	3.9539	3841.0470	5052.1825	15.6940	14893.1200	16991.9875

### Prediction

The incidence number, forecasted by ARIMA(3,1,1)(0,1,2)_12_ model, will have a slightly fluctuation from November 2017 to March 2018 (Table 6).

**Table 6:** The prediction value of ARIMA model.

Time	ARIMA(3,1,1)(0,1,2)_12_ Model
Nov.2017	94415.57
Dec.2017	92139.10
Jan. 2018	94569.93
Feb. 2018	85310.10
Mar. 2018	108736.70

## Discussion

The incidence of Hepatitis B in China had declined from 2010 to 2014 and risen up in recent years. Large population base of China makes it a large infected population [24] and an increased social financial burden [25], even with low incidence rate. Incidence prediction may be of great significance for the prevention and control of hepatitis B before it’s outbreak. Two of the most commonly adopted models in infectious disease prediction were compared in this study and tested their feasibilities in fitting and forecasting hepatitis B in China. The results showed that ARIMA (3,1,1)(0,1,2)_12_ model had higher prediction performance than GM(1,1) model and was more appropriate in forecasting hepatitis B.

Different principles of these two models results in different performances. Structured modeling basis and acceptable forecasting performance make ARIMA model widely used in time series prediction [26]. The model transforms the influence factors of disease into some special time variables and then matching. Periodicity and long-time trend are considered in repeatedly recognition and fitting to determine the optimal model. While GM(1,1) model uses a single variable first order to acquire high prediction accuracy. Besides, the sample size of ARIMA model should be 30 at least, while GM(1,1) model could develop a model with just 4 numbers. Incidence of hepatitis B is influenced by temperature, social economic status, accessibility of medical service and so on. An obvious periodicity of hepatitis B was seen, of which might be more applicable with ARIMA model.

Less requirement of data and easier expression make GM(1,1) model widely adopted in small sample size and uncertain time series predictions. The model is quite susceptible to external influencing factors which may reduce the prediction accuracy in this study. In addition, the prediction length of GM(1,1) model is limited by the quality and length of time series, less than three could be predicted by uncertain time series. Incidence from June to October 2017 were predicted in this study and this may be responsible for low prediction accuracy of GM(1,1) model. Besides, data type also influences the accuracy. A smoother and exponential growth data contributes higher accuracy [27]. Modified grey model is another one which aims at high accuracy [28, 29]. This suggests that modified grey model could be adopted to in hepatitis B prediction.

Model application makes great sense in decision making and was shown useful in disease control. An advanced model could enhance our understanding of population- and individual-level disease dynamics. According to the results, the incidence of hepatitis from November 2017 to March 2018 will increase slightly followed by a sharp decrease, which is similar with usual situation. The incidence of March 2018 (N = 1087367) will be lower than that in March 2017 (N = 110717), but higher than that in March 2016 (N = 105745) and March 2105 (N = 104427). This result indicated that more effective strategy should be established before March 2018 to prevent HBV infection rise again. Disease regulators need well preparation before a peak period of disease, such as prevention and control measures, formulate a management strategy and be careful for disease outbreak. Self-preservation also makes great sense. Unsafe sexual behavior, iatrogenic infection and HBV infected blood exposure should be avoided.

There are some limitations in this study. First, the data of this study came from the government report. Monitor data was influenced by the intention of infected person. Some factors may weaken their test willingness such as poverty or poor medical condition. Thus we assumed that the monthly report data in this study may less than actual incidence of hepatitis B slightly. This study aims to provide a reference for model selection of hepatitis B prediction and far more accurate model should be studied. Second, GM(1,1) model requires undulate or less fluctuate time series. The model is commonly applied to annual prediction but seldom adopted in nationwide monthly incidence prediction. Only ten months incidence data was collected to develop the model. No periodicity was seen in a year and this is suitable for GM model, but this may increase the prediction error and annual data or less fluctuation data may help improving prediction performance. Finally, only variation of hepatitis B incidence with time was considered, the function of other possible impacting factors were ignored such as medical conditions and environment. Thus, data should be continually update to ensure high prediction accuracy and give an accurate warning before hepatitis outbreak [30].

## Supporting information

S1 File The data of hepatitis B incidence in China from March 2010 to October 2017. (DOC)
