# Supplementary material for: Comparison of ARIMA and GM(1,1) models for prediction of hepatitis B in China
Source: PLoS One. 2018 Sep 4;13(9):e0201987. doi: 10.1371/journal.pone.0201987 (PMC6122800; doi:10.1371/journal.pone.0201987)
Supplement: S1 File — (DOC) [file pone.0201987.s001.doc]

S1 Table The data of hepatitis B incidence in China from March 2010 to October 2017

|  | 2010 | 2011 | 2012 | 2013 | 2014 | 2015 | 2016 | 2017 |
| --- | --- | --- | --- | --- | --- | --- | --- | --- |
| Jan. |  | 100932 | 89614 | 102367 | 90210 | 96649 | 89699 | 86657 |
| Feb. |  | 85649 | 123239 | 78884 | 83068 | 72869 | 82204 | 99417 |
| Mar. | 117048 | 117553 | 124899 | 107535 | 99292 | 104427 | 105745 | 110717 |
| Apr. | 104691 | 106464 | 107415 | 97225 | 94768 | 94350 | 93190 | 98123 |
| May | 100653 | 106263 | 113147 | 96978 | 91936 | 91194 | 95079 | 101783 |
| Jun. | 98691 | 103219 | 101592 | 86335 | 88201 | 89224 | 90166 | 100155 |
| Jul. | 105007 | 108259 | 108079 | 97401 | 95648 | 93586 | 91219 | 98501 |
| Aug. | 103030 | 113093 | 107139 | 97473 | 94075 | 89228 | 97670 | 103977 |
| Sept. | 93643 | 100517 | 94868 | 87915 | 87827 | 89806 | 87390 | 96856 |
| Oct. | 89066 | 99591 | 95678 | 86161 | 85996 | 86393 | 85480 | 89105 |
| Nov. | 101234 | 107208 | 100209 | 88479 | 85125 | 87284 | 91478 |  |
| Dec. | 97590 | 103488 | 91441 | 87609 | 88397 | 90103 | 91371 |  |
